# Indents

**Published:** 1909-11-15

**Authors:** 


### INDENTS.

☞ **Brief Articles of Technical or Scientific Value will receive notice and comment. Contributions invited.**

*Pickling Vulcanite Plates.* Pack the rubber so carefully that a uniform thickness is obtained; vulcanize rather a little too much than just right. Wash carefully, and with a fine spatula remove all the particles of plaster that may adhere to the plate. Then mix in a wide glass equal parts of commercial nitric acid and sulphuric acid, and immerse the piece for one-quarter of a minute. On the surface of the mixture a coat of foam of the color of the rubber used will appear, which after the piece is withdrawn with a wooden stick will be seen to cover the whole piece. After this foam has been washed and brushed off in plenty of water, the plate is dropped once more into the liquid and allowed to remain another one-quarter of a minute. If after washing some imperfection is still noticed, a third acid bath and washing is applied. The plate is then dried and submerged in ammonia and subjected to long and thorough brushing. This removes all traces of acid and any coloring matter due to the pickling. After freely washing with soap, the piece is ready to be placed in the mouth. The great advantage over the old tin-foil method is that the reproduction of the oral tissues is a much more perfect and exact one.—H. Leger-Dorenz, *Le Laboratoire et Le Progres Dentaire.*

*A Physician May Not Practice Dentistry.* The Supreme Court of Minnesota in a recent decision holds that a person licensed to practice medicine may not by virtue thereof practice dentistry. In the case decided a physician extracted two teeth for one of his patients, took an impression of his patient's mouth and of some cavities, and sent them to a dental laboratory where artificial teeth, etc., are made, and when the finished work he directed to be made from them was returned to him he tnserted the same in his patient's mouth, charging a fee iherefor. The court in passing on this case ruled that a physician and surgeon could operate and prescribe in so far as it is a direction of medical science to the prevention, modification or removal by medicinal and hygienic remedies of the cause and effects of diseases in the dental organs. He may apply his surgical skill to the relief of staphylorraphy, or fracture of the jaws; this is simply oral surgery, and involves only such knowledge as every surgeon must possess. The preparation of cavities of decay in the dental organs for the reception of fillings, and the insertion of fillings, and fitting artificial teeth to a patient's mouth, require special knowledge that is neither included in either medical or surgical science, but belongs especially to dental science.—*Journal A. M. A.*

*The Museum of the Odontological Section of the Royal Society of Medicine*

The Museum of the Odontological Section of the Royal Society of Medicine, at present contained in the premises of the Society in Hanover Square, has been offered to and accepted by the Council of the R.C.S. as a trust. The transferrance of this important collection represents one of the largest and most valuable additions ever made to the Museum of the Royal College of Surgeons. The collection contains about 5,000 specimens arranged in 113 large mahogany cases. At the present time the College Collection of Odontological specimens, many of them being those used, by Hunter in the preparation of his monograph on the teeth, are shown in several sections of the Museum. It is proposed that these should be brought together and added to the collection now entrusted to the College, the whole to be arranged in a commodious and accessible room in the basement of the Museum—under room II., to be known as the

"Odontological room." The addition of this collection, although it will entail a considerable expenditure of both money and labour, will place the Museum of the Royal College of Surgeons in possession of one of the most valuable odontological collections in the world.—*Dental Record.*

*Bismuth Paste in he Treatment of Pyorrhoea.* Dr. Rudolph Beck, of Chicago, recommends for injecting pyorrhoea pockets a paste made by the following formula:

| | |
|---|---|
| Bismuth subnitrate | 30 parts |
| White wax | 5 parts |
| Paraffin | 5 parts |
| Vaselin | 60 parts |

Mix while boiling.

This he injects into the pockets with an all-metal syringe with a fine, tapering flexible point. The syringe should hold about half an ounce of the paste. The first injection he makes before removing the deposits. The next day he removes all deposits and reinjects the paste, and repeats the injections as frequently as may be required. In beginning the treatment of pyorrhoea he uses the injection every other day until signs of improvement are noticed, then less frequently. It stops pus formation, and quickly brings about a healthy condition of the parts. He uses the same paste in treating dento-alveolar abscess, endeavoring to fill the sack, and the fistula thoroughly. He directs attention to the possibility of bismuth poisoning, but does not think it is to be anticipated from the small amount used.—*Dental Review*, January, 1909, page 1. (Injecting bismuth subnitrate into the intestines as an aid in X-ray diagnosis has been followed by serious results.—ED. *Dental Brief.*)

*The Chemical Composition of Teeth.* Gassman has made a chemical analysis of human and of dogs' teeth, in order to ascertain whether there is any considerable difference in their composition. The results, which are recorded in the *Zeitschrift fur Physiologische*

*Chemie,* 1908, vol. lv., p. 455, are given in percentages in the following table:

| Human Teeth— | Water | Loss on calcination | Calcium |
|---|---|---|---|
| Canines | 8.00 | 22.20 | 29.78 |
| Deciduous | 3.76 | 22.84 | 29.59 |
| Third molars | 6.91 | 18.33 | 31.65 |
| Aged persons | 8.17 | 21.42 | 30.25 |
| Dogs' teeth | 10.97 | 25.00 | 27.33 |

These figures show that human teeth contain more calcium than those of the dog, and the third molars, which are richest in calcium, contain nearly 5 per cent. more than is present in dogs' teeth. The loss due to calcination affords an approximate indication of the amount of organic matter present. It will be noticed that whereas dogs' teeth are the richest in organic matter, human third molars are poorest in that respect. The quantity of phosphoric acid is proportional to the calcium present. Human teeth are richest in chlorin and the salts of potassium, whereas in dogs' teeth the salts of sodium predominate. It is noteworthy that the third molars, which are the most prone to decay, are relatively rich in calcium and poor in organic matter.—*British Dental Journal.*

*Grinding Edge Tools.* Grindstones are usually used wet for the following reasons: Friction produces heat and the friction of the metal on the stone, if the stone is driven at a high speed will heat the edge of the tool enough to draw the temper. The water, acting as a lubricant, prevents this. The water also helps to keep the stone clean by preventing the fragments of steel ground off clogging between the grains of the stone. A wet stone cuts quicker than a dry one, but the surface is not as fine as in dry grinding. Oil is used on the finishing stone for the same purpose; it makes the tool slide more easily on its surface, while the grains of silica, which really do the the work, project through the film of oil. Oilstones should be mounted on a wooden block with a cover to keep the dust off. Neatsfoot or olive oil is considered the best. If necessary to thin it, use paraffin oil; never use turpentine, as it causes the oil to dry quickly. Glycerine is a good substitute for oil, and is less liable to thicken on the stone. In spite of all care, oilstones will wear to an uneven surface. To make it level again, clean off all traces of oil and rub on a flat stone with plenty of water. A sheet of glass paper placed on a level surface does very well. If the stone is a very hard one it may be necessary to use emery or carborundum, starting with a coarse and finishing with a fine grade. New tools should be left sufficiently large to permit the skin of steel decarbonized in forging to be ground off. The extra labor will be well paid by the superior quality of the finished tool. Always remember that dull tools do not cut either easily or quickly, and do not leave a clean, smooth surface. Time spent in keeping tools in good order is time well spent.—M. Cole, *Electrician and Mechanic.*

*Flies.* "We have had previous occasion to call to the common-sense methods of educating the people in health matters adopted by the Department of Health of the City of Chicago, notably in connection with an excellent little leaflet bulletin issued fortnightly for public instruction. We have now received from the same bureau a small but striking poster which is headed "Speaking of Flies" and which proceeds to speak of them in terms of the ungarnished truth. And lest the verbal recital of Musca's potentialities for evil should not seize the general imagination, a pictorial border (designed, we read, by the Florida State Board of Health) is introduced showing plagues of flies pursuing their abominable courses. In the left-hand column of this border are shown a consumptive's spittoon, a garbage-can, a muck-heap, a dead dog, and a privy mid, den, each with its attendant swarm of house-flies. These-grown to disgusting dimensions, are shown flitting across the upper and lower borders of the sheet and alighting upon the various articles of food depicted in its right-hand margin, which includes a couple dining amidst a cloud of the domestic pests and a fly-ridden sick man in a bed labelled "typhoid." As a counterfoil to these "horrible revelations" there is printed some practical advice on, "What to do to get rid of flies," which consists largely of procedures often advocated in these columns, the screening of food and windows, the use of fly-traps, or of formaldehyde, or of bichromate of potash solution. The burning of pyrethrum powder is also recommended, and instructions are given for the elimination of the breeding places of flies. Americans have the reputation at least of being a thoroughly "practical" nation, and we can hardly suppose that they will read this poster and altogether ignore its warnings. We hope the poster will come to the notice of public health officers in this country.—*Dental Record.*

*Oral Prophylaxis.* I have seen teeth in the mouths of patients in the office of Dr. D. D. Smith, where the eight anterior teeth, after years of treatment, presented flat surfaces instead of convex surfaces. The caries at the gingival line had been polished, ground away, and had received prophylactic treatment. Those teeth were not sensitive at all. After polishing I paint the surface with chloride of zinc, as I think it reduces the sensitiveness of the dentine. The deliquesced solution is used and repeated often.

We have listened to remarks this evening on the mechanics of filling teeth by very learned authorities on the subject. I think "oral prophylaxis" is so far ahead of the mechanics of filling teeth that there is no comparison between the two. Reference was made to establishing a dental institnte for the benefit of the public. If, in our colleges, there could be established a chair devoted to the teaching of Oral Prophylaxis, it would do more good for our dental students, and indirectly the public, than any other method of teaching.

As to brushing the teeth, I think a liquid alkaline soap solution is as good as anything, except in certain mouths which seem to require a heavy grit; some demand more grit than others. I think a pure antiseptic *liquid* soap, with an alkaline reaction, is a good means of establishing cleanliness.

As to the sensitiveness in working on teeth in this locality, I believe in many instances it would be a good plan to treat the teeth once a month, or once every two weeks, if necessary, by the prophylactic method, and after two or three months of such treatment one can work on those cavities without the patient suffering greatly. I began treatment in a case of pyorrhea about a year ago, the patient having a great deal of pus in the mouth and saliva highly acid. At the time the work was begun one could not touch the teeth with a cold instrument, on account of the sensitiveness. The other day I took a bur, burred into a sound tooth and took the pulp out for capping in bridge work. There was no sensitiveness until the live pulp was nearly reached. This shows what environment means. Some time before this one could not touch the outside of the teeth. This case demonstrates the effectiveness of prophylaxis.—Sidney McCallin, in *Review.*

*Electro-Deposition for Platinizing Bridge-Saddles and Arches.* Frequently under the most perfectly constructed gold saddles a slight irritation of the gingivæ is observed, while under platinum the conditions remain always normal. Platinum is, however, much more difficult to swage than gold. This difficulty the author has avoided by first swaging a saddle of platinum plate of 0.15 mm. thickness, to which he soldered a second saddle of 22-karat gold. As this procedure is complicated by considerable difficulties, the author chanced upon the idea of building the bridge of gold and of subsequently platinizing by electro-deposition the portions which are to come in contact with the gingivæ. The following formula yielded excellent results: 5.8 grams of platinum give 10 grams of platinum chlorid. The platinum is rolled out as thinly as possible, cut in small pieces, and dissolved in about 50 cubic centimeters of aqua regia. The excess of acid is driven off in a water-bath. Platinum is dissolved in boiling aqua regia, which during its evaporation must be continually renewed until all the platinum is dissolved, when the aqua is entirely driven off. Finally a dry crystal mass of yellow or brown color remains, which is taken up in distilled water. Solution 1: Aqua destillata 1 liter, ammonium phosphate 20 grams, phosphate of soda 100 grams, platinum chlorid 4 grams. Solution 2: four grams of platinum chlorid in 1 deciliter of aqua distillata and 20 grams of ammonium phosphate in 2 deciliters of aqua destillata. Both solutions are poured together under stirring, when a yellow precipitate of platinum-sal-ammoniac is noted. To this mixture a third solution is added: 100 grams of phosphate of soda in 7 deciliters of aqua destillata, and the whole is boiled until the precipitate has disappeared and on stirring no odor of ammoniac is noticeable. At first the evaporating water is replaced; after the ammoniac odor has disappeared, the whole mixture is condensed to about one and one-half liters. The platinizing is done under heat, though even at tepid temperature excellent results may be had. This is important, since, if only portions of the bridge are to be platinized, the other parts must be covered with sticky-wax. By this method the most complicated saddle can be finished in less than half the time required for swaging it in platinum plate, and in ten minutes the desired portions are covered with an absolutely solid and beautiful layer of pure platinum.—Dr. Wilhelm Thiersch in Sept. *Cosmos.*

*Brushing the Teeth.*

The following are extracts from letters appearing lately in the *Daily Express* —"I am the oldest of seven brothers. I have cleaned my teeth regularly; they have not. I have never lost a tooth; they have."

A. CHAPMAN.

"I think the habitual use of toothpicks of far more importance than the use of the tootbrush. In countries in the South of Europe, for instance, where you see finer specimens of natural teeth than we can often boast of, toothbrushes among the poor and the peasantry are unknown. In Portugal, where good teeth prevailed some years ago, toothpicks were used most scrupulously even among the numerous beggars. Brushing the teeth does not always entirely clean them even when carefully and skillfully done."

H. B.

"I am really disheartened, not to say disgusted, that at the present stage of the world's progress there are still people who don't clean their teeth." J. F. LEGARD.

"One need only inspect the teeth of children in any orphanage or other institution where a dentist attends and toothbrush drill is considered as important as washing one's face, to be convinced by the appearance and soundness of the teeth of the children." JOHN BUTTON.

"Nature herself is sufficient in the rearing of her creatures if her laws are obeyed. Animal's teeth are not 'surgically cleaned,' senor do they need it. Let us, therefore, revert to natural and wholesome food, and then I think we shall hear less about physical degeneration. Would it not be better to urge a stricter enforcement of the Food and Drugs Act, and if necessary extend them, instead of adding to the already heavy burden of rates by universal free dentistry?" H. ARKLE

"The cause of bad teeth is 'cleaning.' Other animals do not clean their teeth, why should man? It is contrary to nature. Did Adam and Eve or Methuselah use a toothbrush? My yacht steward never did. He is forty-five years of age and never had a toothache. Destroy every toothbrush in the land, and in ten years every dentist would be in the workhouse." DENS.

"If money need be spent the first expenditure should be to decide the cause of the increase of dental caries. The increase of dental caries is not universal, as in certain districts teeth are now as sound as ever. For instance, many French peasants have sets of teeth with no sign of dental caries, and they use no toothbrushes." THOMAS G. READ.

*The Illinois Code of Ethics.* At the recent meeting of the Illinois State Dental Society a new code of ethics was adopted by the Society to take the place of the one so long in use. The old code has done great good to the profession and there is nothing but respect for its teachings, but it was felt that some of its provisions should be made more specific and that there should be some additions made to its restrictions. The new code reads as follows:

SECTION 1. In his dealings with patients and with the profession, the conduct of the dentist should be in accordance with the Golden Rule, both in its letter and its spirit.

SEC. 2. It is unprofessional for a dentist to advertise by handbills, posters, circulars, cards, signs, or in newspapers or other publications, calling attention to special methods of practice, or claiming excellence over other practitioners, or to use display advertisements of any kind. This does not exclude a practitioner from using professional cards of suitable size with name, titles, address and telephone number printed in modest type, nor having the same character of card in a newspaper. Neither does it prevent a practitioner who confines himself to a specialty from merely announcing his specialty on his professional card.

SEC. 3. It is unprofessional for dentists to pay or accept commissions on fees for professional services, or on prescriptions or other articles supplied to patients by pharmacists or others.

SEC. 4. One dentist should not disparage the services of another to a patient. Criticism of work which is apparently defective may be unjust through lack of knowledge of the conditions under which the work was performed. The duty of the dentist is to remedy any defect without comment.

SEC. 5. If a dentist is consulted in an emergency by the patient of another practitioner who is temporarily absent from his office, the duty of the dentist so consulted is to relieve the patient of any immediate disability by temporary service only, and then refer the patient back to the regular dentist.

Sec. 6. When a dentist is called in consultation by a fellow-practitioner he should hold the discussion in the consultation as confidential, and under no circumstances should he accept charge of the case without the request of the dentist who has been attending it.

Sec. 7. The dentist should be morally, mentally and physically clean, and honest in all his dealings with his fellow-man, as comports with the dignity of a cultured and professional gentleman.

It will be seen that this code contains provisions which have not previously been found in any dental code.

Section III is a conspicuous instance of this, and it is so specific that there is no possibility of mistaking its meaning. It is striking a somewhat prevalent evil at its very root, and the result will place our profession on a higher ethical plane than ever before. The unanimity with which this code was adopted by the state society is an indication of the rapid advance being made in ethical standards on the part of the members, and it is hoped that the practical working of the code will prove beneficial to all concerned.—*Dental Review.*

*Dentistry in Austria.* The conflict in Austria between the dental surgeons and the dental mechanics has lately assumed the character of a battle, in which qualified and unqualified practitioners are struggling for supremacy, and it therefore deserves the careful attention of the entire medical profession. In that country the practice of dentistry is restricted to the dental surgeons, who must possess an M.D. degree, and must have also passed certain dental examinations. The progress of artificial dentistry, especially the art of crown-bridge work and regulation of the teeth, has brought into existence a large number of assistants to the dental surgeons, men without any medical degree or systematic instruction but possessing a good knowledge of the mechanical process employed in artificial dentistry. Ever since dentistry became a lucrative occupation these men have tried to become independent of the surgeons and have encroached upon the domain of the latter until they undertook extraction of teeth, filling of cavities, and other surgical procedures without interference from the governmental authorities or medical corporations. Growing bold by degrees they have been endeavoring to have the practice of dentistry freed from the existing restrictions and thrown open to all who belong to the "guild" of dentists. The dental surgeons have naturally taken active steps in defence of their rights; and in the vicissitudes of this strife the governmental decisions have been partly for and partly against the medical men. At last a compromise was suggested by the unqualified dentists namely (1) that dentistry should in future be practiced only by men holding the degree of M.D., and that no new dentists should be admitted to the "guild," so that this class would in course of time die out, leaving the field to the qualified men; and (2) that the dental mechanics should meanwhile be entitled to do all work necessary for the fitting of artificial teeth in the mouth. As the second condition meant nothing else but a tacit permission that surgical work might be done by men not having any hospital experience or medical diplomas the compromise was not accepted by the dental surgeons. Having obtained the help of some Members of Parliament the dental mechanics now began to demand a complete separation of the two branches of dental practice; the purely surgical one (filling of cavities and extraction of teeth) they offered to leave to the dental surgeons, whilst for themselves they asked for nothing else but the purely mechanical branch (artificial dentures, crowns, bridges, and regulation of teeth), together with permission to carry out the minor surgical procedures in the mouth nesessary to fit in the dentures, such as the extraction of stumps, preparation of teeth for crowning, etc. This would mean, on the one hand, the absolute ruin of the dental surgeons and, on the other hand, the legalising of surgical practice on a special part of the human body by unqualified men. The whole profession has been roused and a meeting, arranged by all the medical corporations, has taken place in Vienna for the purpose of making a protest. All the speakers in this meeting pointed out the dangers threatening the public from the indiscriminate granting of permission to practice dentistry. As the dental mechanics have brought many influential men over to their side it is not impossible that they may be successful in their endeavors. Concerted opinions by the entire profession must therefore be organized, the public must be informed of the actual facts through the press, and there is still hope that victory may remain with the profession.—*Record*.

